# 

**Published:** 1908-09-12

**Authors:** 


### CONTENTS.

PAGE

**Alcohol and Exaggeration** ... 617

**New Remedies** ... 618

**Annotations—**

Army and Navy Male Nurses—Physiology, Heredity, and Psychology—Property in Ideas ... 619

**Medical Opinion and Movement** ... 620

**Special Articles—**

Nineteen Cases of Sudden Death ... 623

Appendicostomy: the Operation, its Indications and its Results ... 626

**Medicine—**

Enlargements of the Spleen—VI. ... 627

Notes on Clinical Methods ... 628

**Surgery—**

Intussusception: Some Remarks on its Diagnosis and Treatment ... 629

**Life Assurance—**

Practical Hints upon Medical Examinatiou for Life Assurance.—IV. ... 631

PAGE

**Therapeutics—**
Natural Mineral Waters ... 632
**Diseases of Children—**
Sepsis Neonatorum ... 633
**Public Health and Hygiene—**
Milk, Fresh from the Cow ... 634
**The Practitioner's Relaxations and Hobbies—**
Cards and the Doctor ... 635
**Dermatology—**
Relapsing Eczema... ... 636
**Hospital Administration—**
Some Modern Continental Hospitals... ... 637
**News and Coming Events** ... 639
**Nursing Administration—**
The Domestic Staff—III. ... 641
**Editor's Letter-Box** ... 642

